# Characterization of PM_10_ and Impact on Human Health During the Annual Festival of Lights (Diwali)

**DOI:** 10.5696/2156-9614-8.20.181206

**Published:** 2018-12-01

**Authors:** Rajyalakshmi Garaga, Sri Harsha Kota

**Affiliations:** 1 Department of Civil Engineering, Indian Institute of Technology Guwahati, Guwahati, India; 2 Department of Civil Engineering, Indian Institute of Technology Delhi, New Delhi, India

**Keywords:** Fireworks, PM_10_, bacteria, sound level, health study, risk level

## Abstract

**Background.:**

Diwali is a Hindu holiday observed each autumn in India, where it is known as the ‘celebration of lights’. Burning of fireworks on this day leads to air and noise pollution, causing adverse effects to human health.

**Objectives.:**

To monitor and analyze air quality and noise levels in a residential college campus in northeast India over Diwali 2015.

**Methods.:**

Components of PM_10_, including metals (cadmium (Cd), cobalt (Co), iron (Fe), zinc (Zn) and nickel (Ni)), ions (calcium (Ca^2+^), ammonium (NH_4__+_), sodium (Na^+^), potassium (K^+^), chloride (Cl^−^), nitrate (NO_3−_) and sulfate (SO_4_^2−^)) and bacterial counts were studied for a period of ten days in November 2015. In addition, a health-based survey of patients attending the institute's hospital during those days was conducted to evaluate the risk level due to fireworks burning.

**Results.:**

The mean PM_10_ concentration during Diwali, 311 μg/m^3^, was 81% higher than other days and 3.1-times higher the Indian National Ambient Air Quality Standards. While noise levels were increased by 65%, the concentration of bacteria in PM_10_ was reduced by 39% during Diwali compared to other days. The concentrations of metals, cations and anions were increased by 51%, 72% and 77%, respectively. A health study conducted during the analysis period revealed an increase in hospital admissions in the campus due to respiratory symptoms. The higher concentrations of metals during the Diwali period resulted in a 0.5% increase in the hazard index.

**Conclusions.:**

The present study suggests that reducing fireworks during Diwali could reduce pollutant concentrations and result in potential health benefits.

**Participant Consent.:**

Obtained

**Ethics Approval.:**

The study and survey design were approved by the Institutional Bioethics Committee of the Indian Institute of Technology, Guwahati.

**Competing Interests.:**

The authors declare no competing financial interests

## Introduction

Concentrations of regulated air pollutants exceed Indian National Ambient Air Quality Standards (NAAQS) in most cities in India.^1^,^2^ Burning of fireworks leads to the release of large amounts of harmful gases and toxic substances into the atmosphere, thereby contaminating the air, causing adverse effects to human health and worsening air pollution. A rapid increase in pollutant concentrations due to fireworks has been previously reported.^3^ Determination of PM_10_ (particulate matter with aerodynamic diameter < 10 μm) in ambient air is essential, as pollution due to fireworks causes pulmonary effects, with greater impacts to the health of young children, elderly persons and pregnant women.^4^,^5^ Fireworks are made up of various organic and inorganic chemicals which create smoke plumes that contain charcoal, sulfur, potassium, lead, aluminum, iron and barium nitrate.^6^,^7^ Chemical reactions propel fireworks into the air and cause them to explode. While sodium (Na^+^) and potassium (K^+^) are used as metal oxidizers, zinc (Zn) is used to produce smoke effects and strontium (Sr) is used to stabilize fireworks.^7^

Pollutants released from fireworks at a higher altitude are diluted before coming into contact with human populations, which can reduce health impacts.^8^ However, ground level firework displays have an immediate impact on human health. Acute eosinophilic pneumonia has been reported in a patient due to inhalation of smoke continuously for three nights from burning of fireworks.^9^ While fever, cough and dyspnoea are often reported initially as acute effects, pollution due to fireworks causes chronic respiratory and cardiovascular disease, pulmonary effects, premature death and cancer.^10−12^ A 30% to 40% increase in the incidence of wheezing, respiratory disease, exacerbation of bronchial asthma and bronchitis has been reported during a Diwali celebration in India.^3^

Several studies have been carried out in different parts of India to assess the impact of fireworks on PM_10_-related air quality.^11^ A short-term study reported a 2–3 times elevation in PM_10_ concentrations during a Diwali festival in Hisar city in northwest India.^13^ PM_10_ during Diwali in Lucknow in northern India was reported to be 7.53-times higher than on other days.^12^ A five-fold increase in PM_10_ concentration was observed in Kolkata in eastern India during Diwali.^14^ A 35-fold increase in PM_10_ concentration during Diwali was reported in Vadodara in western India due to burning of fireworks.^11^ In Rajnandgaon in central India, PM_10_ concentration during Diwali showed a nearly 3-fold increase compared to other days.^15^ A 4- to 10-fold increase in PM_10_ concentration was observed during Diwali in Nagpur in central India.^16^ Owing to already high PM_10_ concentrations, several studies were conducted in the national capital, New Delhi. High concentrations of PM_10_ (767 μg/m^3^ and 620 μg/m^3^) were observed during the Diwali festivals of 2008 and 2009, respectively.^17^ A community-based health survey conducted during Diwali in 2013 revealed an increase in the number of patients in Delhi with problems related to respiratory diseases, hearing issues, eye irritation and headache.^10^

A previous study in northeast India observed an increase in the concentrations of metals, anions and cations during festival days compared to other days.^18^ In Raipur, ion concentrations were 10 times greater than other days.^19^ A similar increase in the metals associated with fireworks burning were reported in Delhi.^20^ The prevalence of several respiratory symptoms such as asthma, inflammation, irritation, and fatigue due to bioaerosols have been previously reported.^21^,^22^ However, studies have observed a negative correlation between bacteria and heavy metals.^23^ Therefore, along with chemical analysis, characterization of bioaerosols is helpful to determine the impact of heavy metals released due to burning of fireworks on bacterial activity.

**Abbreviations**
*A/C*: Anion to cation
*HI*: Hazard index
*NAAQS*: National Ambient Air Quality Standards
*PM*: Particulate matter

In addition to air quality, noise levels are also affected by fireworks burning during Diwali, causing interference in communication and adverse health effects.^4^ In Uttarakhand, 29.6% and 18.1% increases in average noise level on festival days compared to non-festival days were recorded in residential and commercial areas, respectively.^24^ In Chennai, noise levels were considerably higher in residential areas during the Diwali festival.^25^

Only two studies have been reported from northeast India. In Tezpur, a mean PM_10_ concentration of 87.45 μg/m^3^ was observed during festival days, which was 2.13 times greater than normal day concentrations.^18^ Another study from Dibrugarh reported PM_10_ and PM_2.5_ concentrations during Diwali of 168 and 160 μg/m^3^, respectively, which was 5.33 and 5.74 times higher than normal day concentrations.^26^ However, no examination of health correlations or risk levels was carried out in those studies.

Over the years, celebrations of Diwali have grown. However, there have been few studies examining the direct environmental impact of fireworks on the health of residents in a residential campus. Moreover, no investigations have been carried out in the biggest city of northeast India i.e. Guwahati, which has severe air quality issues. The main aim of the present study is to assess PM_10_ and its associated health risk on residents during Diwali, along with noise levels and biological characteristics of PM_10_.

## Methods

**Figure 1:**
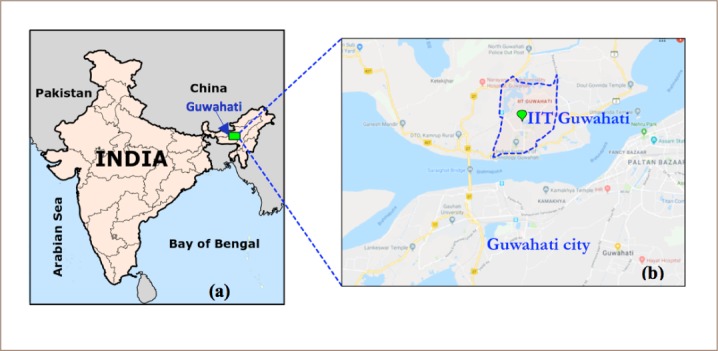
(a) Guwahati city in India; (b) Study area – Indian Institute of Technology Guwahati

The present study was conducted at the Indian Institute of Technology (IIT) Guwahati. Guwahati is one of the fastest growing cities in India and the largest city in northeast India. The institute is located 20 km from the heart of the city. Guwahati is located in Assam, in northeast India, as shown in Figure 1. The city experiences a significant monsoon period, with cold, dry winters from late November to February.^27^ The sampling location is close to an industrial region with an export promotion industrial park, Guwahati biotech park, skin and health care industry, pharmaceutical company, machine industry and a liquid petroleum gas bottling plant. Moreover, construction activities are prevalent throughout the year due to capacity expansion within the premises of the residential area. Vehicle traffic is mainly comprised of motorcycles and passenger cars, with few heavy commercial vehicles used in construction sites.

### Sample collection

A fine particulate sampler, Envirotech APM 550, with an average flow rate of 16.7 L/min, was used to measure PM_10_ concentrations. This instrument works on the principle of impaction to separate particles greater than 10 μm aerodynamic diameter. Filters with 47-mm diameter Whatman^®^ quartz membrane filters and pore size of 0.2 μm were used to collect the samples. The same filters were analyzed for PM_10_ and all other chemical and biological analyses by dividing the sample filter into aliquot parts. For the quality check, filters were sterilized by autoclaving at 121°C (250°F) and 100 kPa (15 psi) above atmospheric pressure for 15 minutes to ensure zero microorganisms before use in the sampler. Filters were carried to and from the site in sealed polyethylene bags for gravimetric analysis and utmost care was taken to avoid handling errors. For further quality assurance, blank samples were analyzed, and necessary corrections were included. Filter papers were desiccated for 24 hours before and after sampling to minimize errors due to moisture and achieve accurate measurements. Samples were weighed with an electronic balance (Model-Kerb & Sohn GmbH) with a sensitivity of 0.1 mg. To prevent manual contamination, a few drops of ethanol were applied to hands during filter placement and retrieval.

Noise level was measured using a sound level meter (Amprobe SM – 20 A) at a height of 3–4 m from ground level, following Indian Central Pollution Control Board guidelines.^28^ A four-day monitoring program was carried out at night during large fireworks displays, from November 11–14, 2015.

Weather data were collected using a weather station (Vantage Pro2) to collect parameters such as temperature, dew point, relative humidity, wind speed and wind direction. This weather station recorded data continuously and the data for each day were retrieved separately. Wind speed measurements from the ground and other obstructions were made at an approximate height of 5 m from ground level.

Sampling was conducted from November 8–17, 2015, to predict air quality variation. Samples collected on November 10, 11 and 12 were treated as Diwali samples. The samples before and after the Diwali period were treated as pre- and post-Diwali samples.

A health-based survey of patients attending the institute's hospital during those days was conducted (Supplemental Material). Information regarding age, body weight and various symptoms such as headache, fatigue, irritation, coughing, sneezing and sinusitis experienced by patients were documented. A total of 350 patients were surveyed, out of which 322 participants with comprehensive information were considered. Patients who had already filled out the forms and returned the next day without any added symptoms were excluded. The chosen 322 participants reported their symptoms on the survey forms issued at the reception center. All the symptoms reported by patients were self-diagnosed. Most participants experienced fatigue or irritation and hence these symptoms were chosen as the limiting threshold for a positive response. However, the response rate was consistent throughout the study period, since this survey was first of its kind for all the residents in the campus.

This information was utilized for further study analyses such as risk level calculation. Participants were informed of the study parameters and assured of the confidentiality of disclosed personal information prior to their participation. The study and survey design were approved by the Institutional Bioethics Committee of the Indian Institute of Technology, Guwahati.

The sampling schedule and meteorological parameters recorded during the study period are shown in Table 1. Data on wind speed and direction were collected every 5 minutes during the analysis period by the weather station and averaged. The dominant wind direction was southeast, with a wind speed of 0.5–1 m/s for 33% of recorded hours.

**Table 1:**
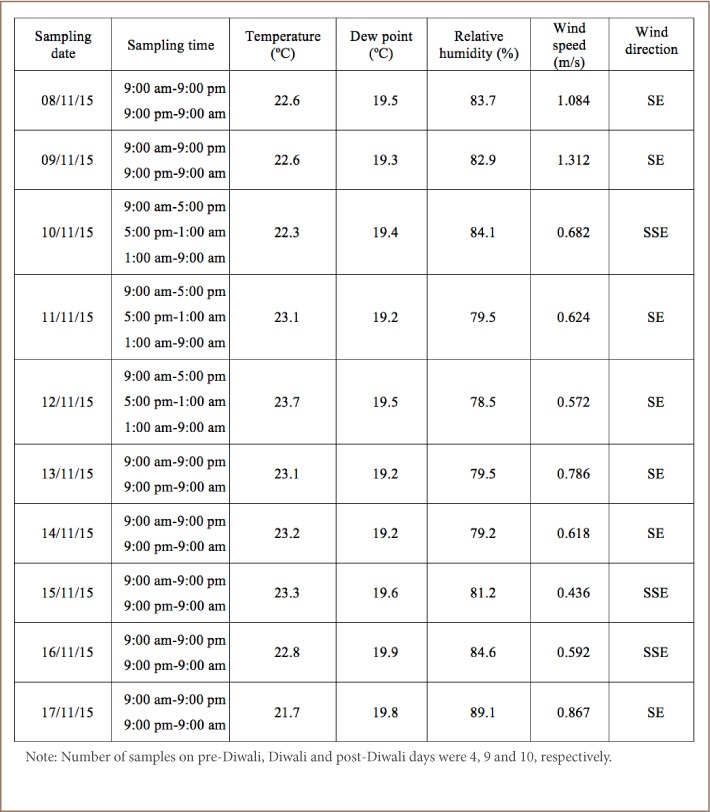
Sampling Schedule and Meteorological Parameters Recorded During the Monitoring Period

### Chemical analysis

PM_10_ collected on each sample filter was manually divided into aliquot parts; i.e. one half and two quarter halves for various purposes. For water soluble ions, an aliquot (1/4th part) of PM_10_ sample was immersed in ultrapure water and ultrasonicated for 20 minutes. The mixture was filtered, and the filtrate volume was adjusted to 15 mL. Samples were stored in prewashed polyethylene bottles and kept at 4°C until analysis in an ion chromatograph (Metrohm 792 basic IC).^18^

An anion column (Metrosep A Supp 5-250/4.0) with a suppressor was used for anions analysis. A solution mixture of 3.2 mM sodium carbonate (Na_2_CO_3_) and 1 mM sodium bicarbonate (NaHCO_3_) was used as the eluent and the flow rate was maintained at 0.7 mL/min. Finally, 50 mM sulfuric acid (H_2_SO_4_) was used for regeneration.

Cations were analysed by a cation column (Metrosep C 4 150/4.0) where a mixture of 1.7 mmol/L nitric acid and 0.7 mmol/L dipicolinic acid was used as eluent with a flow rate of 0.9 mL/min. Twenty (20) μL of sample was measured using inbuilt loop and injected in to the ion chromatograph system.^14^ Calibration and quantification of components were performed using Merck reference standards (CertiPUR) (https://www.merckmillipore.com/IN/en/product/IC-multi-element-standard-VI,MDA_CHEM-109036#anchor_Product%20Information)^47^ of 1, 2, 5 ppm for anions and 2, 5, 10 ppm for cations. All the samples were stored in a refrigerator at 4°C.

For metals, aliquot of PM_10_ sample (1/2 part) was digested in 9 mL HNO_3_ on a hot plate under the operating conditions of 100°C for 2 hours. The filtrate volume was then diluted to 50 mL using ultrapure water and stored in polyethylene vials at 4°C maintaining pH ~ 2. Heavy metals were analyzed using atomic absorption spectroscopy (AAS) (Varian Spectra AA-55).^14^ A metals recovery test was performed using National Institute of Standards and Technology (NIST) standard reference materials for ‘Urban Particulate Matter-1648a’ and showed a recovery rate of 87 to 105% for all the analyzed metals. All dilutions were done using ultrapure water (resistivity 18.2 MΩ.cm). Analysis with the standard solution was repeated ten times and reproducibility tests determined the stability of the instruments.^29^ Every third sample was measured twice to check repeatability. Relative standard deviations were <2% for the concentration of the species measured using the NIST standard reference material. The species minimum detection limits (units: μg/mL) were Cd (0.0028), Co (0.01), Fe (0.060), Ni (0.063), Sr (0.003), Zn (0.018) and Ca^2+^ (0.007), NH_4+_ (0.005), Na^+^ (0.005), K^+^ (0.008), Cl^−^ (0.020), NO_3−_ (0.020) and SO_4_^2−^ (0.020).

### Biological analysis

The aliquot (1/4th part) of PM_10_ sample was dipped in the reagent bottle with sterilized distilled water of 10 mL. The serial dilution technique was followed, and the sample was poured on the petri plate with agar. Bacteria were allowed to grow on nutrient agar medium for 2–3 days incubated at 37°C for all of the samples.^30^ Finally, number of bacteria was quantified and reported as CFU/m^3^.

## Results

**Figure 2:**
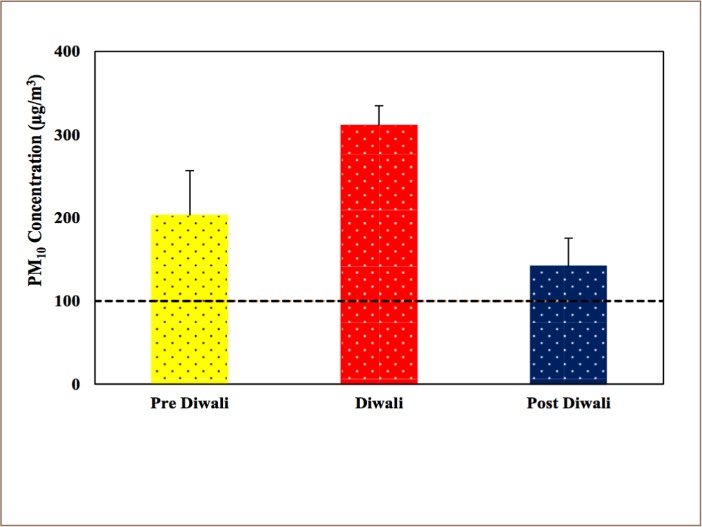
Observed mean and standard deviation of PM_10_ concentrations (μg/m^3^) during pre-Diwali, Diwali and post-Diwali days. Dashed line indicates Indian NAAQS of 100 μg/m^3^

Figure 2 shows the variation of PM_10_ concentrations during pre-Diwali, Diwali and post-Diwali days. As seen in the figure, PM_10_ concentrations always exceeded the 24-hour Indian NAAQS limit of 100 μg/m^3^. The mean concentration during non-Diwali days was 172 μg/m^3^, which were greater than the 24-hour Indian NAAQS limit set by Central Pollution Control Board (CPCB) indicating severe air pollution during fireworks burning.^48^

### Noise levels

The ambient noise level measured during Diwali was 101 dBA. The mean noise level of non-Diwali days was 61 dBA, which is 40 dBA less than during Diwali. In both the cases, the ambient noise level has crossed over the NAAQS limit of 55 dBA, in residential area during day time.^48^

### Biological analysis

**Figure 3:**
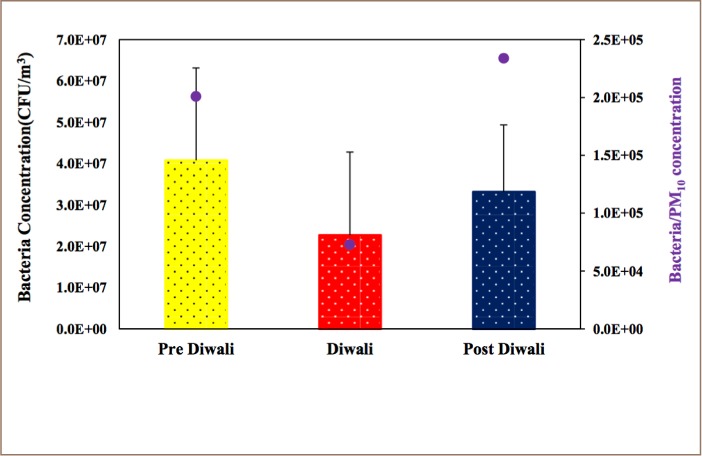
Variation in bacteria concentration (CFU/m^3^) during pre-Diwali, Diwali and post-Diwali days. The ratio (CFU/μg) of change in concentrations of bacteria and PM_10_ during these days is indicated by a filled circle using the right y-axis

Variation in bacteria concentrations are shown in Figure 3. Bacteria concentrations and ratios of bacteria and PM_10_ concentrations were lower during Diwali compared to pre- and post-Diwali days. This could be microorganism inhibition due to heavy metals released in the atmosphere by burning of fireworks. Even though fireworks contain a high mass fraction of carbon content, the major ingredients are metal salts.

### Metals and ionic components of PM_10_

**Figure 4:**
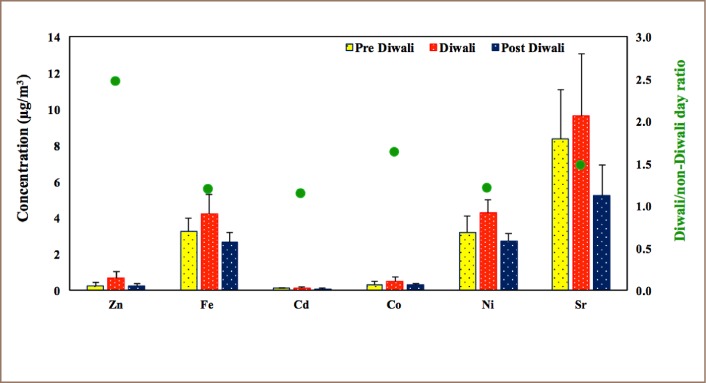
Concentrations (μg/m^3^) of metals (Zn, Fe, Cd, Co, Ni and Sr) during pre-Diwali, Diwali and post-Diwali days. Diwali/non-Diwali day ratios of those metals are indicated by a filled circle using the right y-axis.

Table 2 presents a comparison of PM_10_ and metals concentrations during Diwali in the present study with other studies around the world.^17^,^18^,^20^,^39^,^43^,^44^,^45^ The metal concentrations in the present study, except for Fe and Zn, were higher than in other studies. Concentrations of six metals during Diwali, and pre- and post- Diwali days are shown in Figure 4.

**Table 2:**
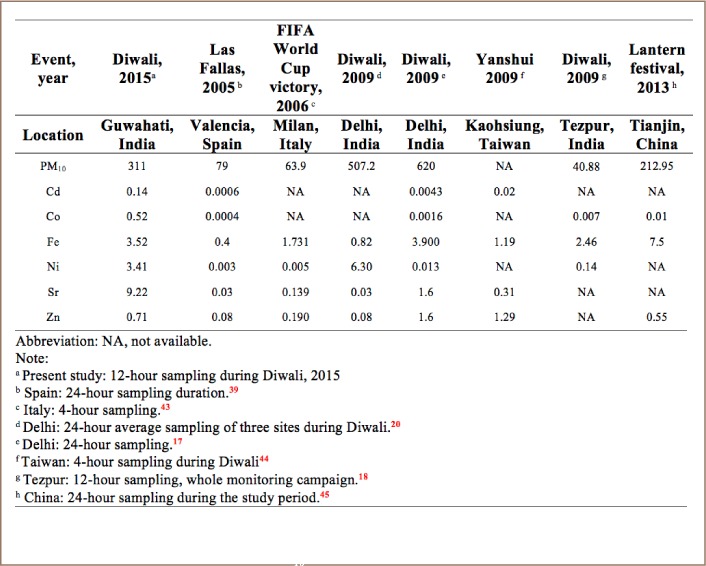
Comparison of PM_10_ and Metals (μg/m^3^) Concentrations During Fireworks Displays Across Studies

**Figure 5:**
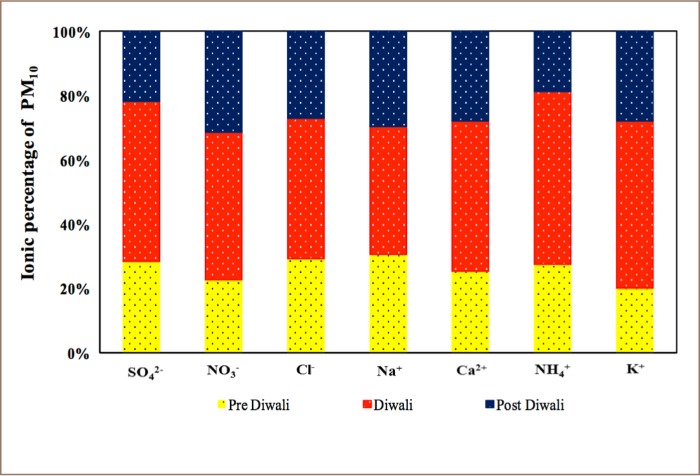
Percentage variation of ions in PM_10_ during pre-Diwali, Diwali and post-Diwali days

Table 3 shows the mean concentrations of water-soluble ions measured during fireworks in the current study compared to other studies around the world. In the present study, there was a decreasing order of anions and cations of Cl^−^ > SO_4_^2−^ > NO_3−_ and K^+^ > NH_4+_ > Na^+^ > Ca^2+^, respectively, during the monitoring campaign. In comparison with other studies in this region, concentrations of studied ions were higher. The study location is a city with more pollution sources, leading to higher PM_10_ concentrations than other studies. Figure 5 depicts the percentage variations of water-soluble ionic species collected during pre-Diwali, Diwali and post-Diwali periods.

**Table 3:**
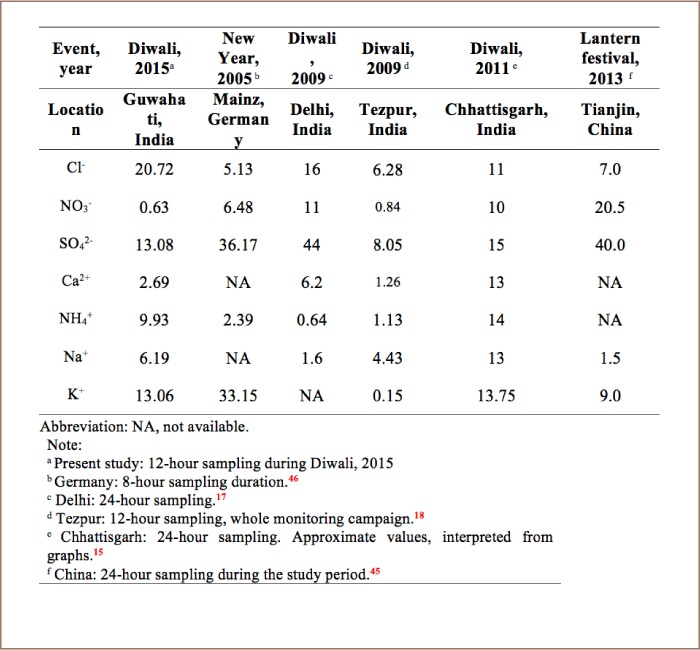
Comparison of Concentrations of Ionic Components of PM_10_ (μg/m^3^) Monitored During Various Firework Episodes Across Studies

### Acidity/basicity of PM_10_

The anion to cation (A/C) ratio gives an insight to the acidic or basic nature of aerosols. In order to calculate ion balance, mass concentrations of ions were converted to micro equivalents (μeq), as shown in Equation 1.^32^

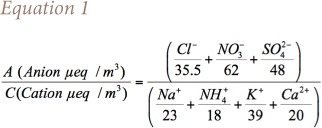



**Figure 6:**
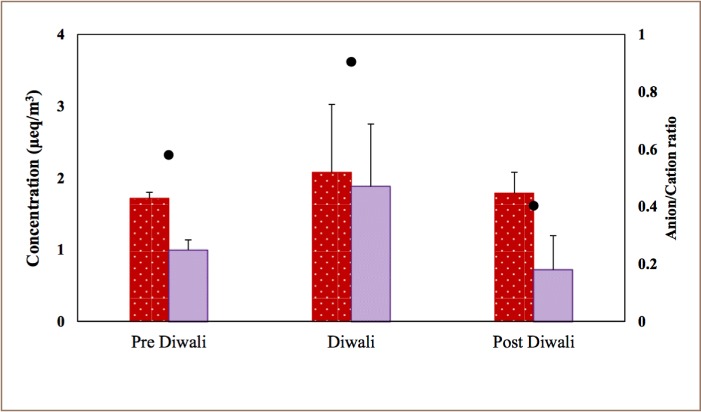
Cation and anion concentrations (μeq/m^3^) and their anion/cation ratios (secondary y-axis) during pre-Diwali, Diwali and post-Diwali days

Figure 6 shows the graphical representation of A/C ratios for the Diwali period along with pre- and post-Diwali periods. An A/C ratio greater than 1 indicates the particle's acidic nature, whereas a ratio slightly less than 1 indicates the contribution of unmeasured carbonate ions.^32^,^33^ A low A/C ratio indicates the basic nature of aerosols.^34^

### Health survey and assessment of health risk due to heavy metals

**Figure 7:**
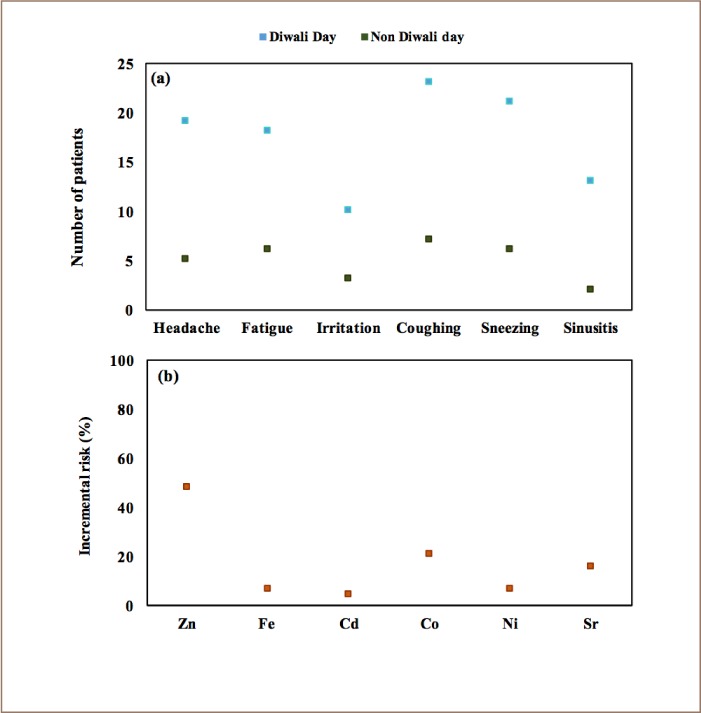
(a) Number of patients reporting headache, fatigue, irritation, coughing, sneezing and sinusitis during Diwali and Non-Diwali days, and (b) Incremental risk (%) due to Diwali from exposure to metals

Panel (a) in Figure 7 indicates that the number of patients reporting headache, fatigue, irritation in eyes, coughing, sneezing and sinusitis increased by 3.8-, 3-, 3.3-, 3.3-, 3.5- and 6.5-times, respectively, on Diwali days compared to non-Diwali days. Similar findings have been found in a post-Diwali morbidity survey conducted in Delhi.^10^

In the present study, five major metals (cadmium (Cd), cobalt (Co), iron (Fe), Zn and nickel (Ni)) were used to assess the possible noncarcinogenic human health risk due to this pollution episode. Two scenarios were taken into consideration. In scenario I, the cumulative hazard index (HI) due to metals (Cd, Co, Fe, Zn and Ni) for an annual period (350 days) was estimated using average concentrations during non-Diwali days. For this calculation, 350 days was based on United States Environmental Protection Agency (USEPA) guidelines which describes this value as the most frequent exposure that is reasonably expected at a site with two weeks of vacation.^35^ The degree of over or under estimation was considered to be negligible.

Scenario II involved two parts (a) and (b). In II (a), the HI was calculated for all the metals for 347 days, excluding the Diwali period, by utilizing concentrations as in scenario I. In II (b), the HI for the Diwali period was calculated. Summation of II (a) and (b) was compared with the HI from Scenario I to determine the excess risk due to Diwali.

The non-carcinogenic health risk to an individual due to exposure to all metals ‘j’ is given by Equation 2:^20^

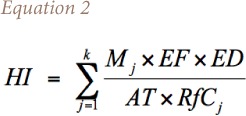
where, RfC is the inhalation chronic reference concentration (mg/m^3^) obtained from the Risk Assessment Information System (RAIS), M is concentration of a metal (mg/m^3^), EF is exposure frequency (days/year), ED is exposure duration (years), and AT indicates averaging time [age (years) × 365 (days/years)].^10^

Using data collected from patients, and concentrations measured during the analysis, the percentage incremental risk (i.e. the percentage increase between Diwali and non-Diwali days) due to each metal is estimated using Equation 3 and shown in panel (b) of Fig. 7.


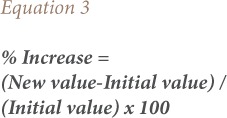


## Discussion

An increase in concentrations was observed for all six metals during Diwali compared to pre- and post- Diwali periods, similar to the findings of earlier studies.^7^,^17^,^19^,^20^,^31^

The mean PM_10_ concentration during Diwali was 311 μg/m^3^ (ranging from 294 μg/m^3^ to 328 μg/m^3^), which is 1.81 times higher than non-Diwali days. The mean concentration during pre-Diwali days was 203 μg/m^3^ (ranging from 163 μg/m^3^ to 264 μg/m^3^), and 141 μg/m^3^ post-Diwali days (ranging from 106 μg/m^3^ to 295 μg/m^3^).

In both the cases, the ambient noise level exceeded the Indian NAAQS limit of 55 dBA set by CPCB for the residential area during the day.^48^

Heavy metals such as lead (Pb), Fe and Zn are known to have potential effects on microbial activity due to their toxic nature, and are the main components in the manufacturing of fireworks.^37^ This could be the reason for the reduced concentration of bacteria during the Diwali period. However, a proper characterization of bioaerosols is required to quantitatively demonstrate this point in the future.

The concentrations of all metals together during the Diwali period were increased 1–2.5-fold compared to the non-Diwali period. This gradual increase in concentrations of metals from pre- to Diwali days, followed by a decrease in post-Diwali days could be due to burning of fireworks. This is also supported by the 2.3-fold increase in Sr, a tracer metal of fireworks burning, which showed a larger increase compared to other metals.^20^

Concentrations of all the ionic species peaked during the Diwali period compared to normal ambient days. Concentrations of Ca^2+^, NH_4+_, Na^+^, K^+^, Cl^−^, NO_3−_ and SO_4_^2−^ were increased by 1.1-, 2.3-, 1.3-, 2.2-, 1.6-, 1.7- and 2-times, respectively, similar to the findings of a previous study in Beijing.^31^ A significant increase in concentrations of K^+^ and Cl^−^ were observed during Diwali period, indicating that the fireworks mixture is comprised of potassium salts, chlorates and perchlorates which are used as oxidizers. Moreover, chloride, nitrate and sulphate are used in different metal salts to produce different colors.^38^,^39^ For example, calcium chlorides and sulphates produce orange flames; and potassium nitrate, potassium chlorate and potassium perchlorate impart a violet-pink color to the sparks, which are a component of black powder, the combustible material.^39^ Therefore, these species are bound to increase when fireworks are burnt in large amounts.

In the present study, NO_3−_/SO_4_^2−^ declined by 95%, indicating a high concentration of SO_4_^2−^ which can be attributed to the release of a large amount of sulfur dioxide (SO_2_) during burning of fireworks. This SO_2_ could be transformed to particulate SO_4_^2−^ through gas phase oxidation by hydroxyl radicals or aqueous phase oxidation involving hydrogen peroxide, ozone, and other oxidants.^14^,^40^ In addition, sulphate salts are mainly used as coloring agents in fireworks, which may be the reason behind higher concentrations of sulphate in PM_10_.

In the present study, during non-Diwali days, the average A/C ratio was found to be 0.45, half the ration found on Diwali days (0.9). Similar observations were made by an air quality study conducted in residential sites in Bhilai.^19^ This indicates that fireworks burning resulted in a more acidic particle composition.

The estimated incremental risk was highest for Zn (1.013), followed by Co (1.005), Sr (1.004) and the remaining elements Ni (1.0017), Fe (1.0016) and Cd (1.0011). The results indicated that the cumulative HI for Scenario II, which includes the Diwali period, showed a risk level increase of ~ 0.5% in health effects due to chronic lifetime exposure to metals alone. It should be noted that this risk level rise was only due to metals, and in ambient settings the risk level will be higher due to chronic exposure to many other pollutants such as SO_2_, mono-nitrogen oxide species (NO_x_) and elemental carbon released by fireworks.

## Conclusions

In the present study, concentrations of metals and ions in PM_10_ were measured for a period of ten days in 2015, coinciding with the Diwali fireworks festival in northeast India. The results indicated that while the concentration of metals increased by 51%, ions increased by 74%. PM_10_ showed a shift from anion to cation deficiency during fireworks burning. Additionally, ambient noise levels measured during Diwali were 101 dB, 40 dB higher than levels on non-Diwali days. Furthermore, the mean concentrations of bacteria during Diwali days showed a 61% reduction compared to non-Diwali days.

The increased level of particulate matter corresponded with a 67% increase in the number of patients attending the hospital during the Diwali period. The chronic lifetime exposure to metals alone during Diwali increased the health risk level by 0.5%. This study stresses the importance of regulated and monitored fireworks burning in regions with a high population density. The present study is limited in external validity due to its relatively small sample size and short duration. However, this study suggested the possible impact of fireworks on human health. Future time series analyses (for example, studies done by Atkinson^41^ and Sahu and Kota,^42^) are needed using data collected from additional fireworks burning events to quantify the relationship between PM_10_ concentrations and health risks.^41^,^42^

## References

[1] Kota SH, Guo H, Myllyvirta L, Hu J, Sahu SK, Garaga R, Ying Q, Gao A, Dahiya S, Wang Y, Zhang H (2018). Year-long simulation of gaseous and particulate air pollutants in India. Atmospheric Environ [Internet].

[2] Garaga R, Sahu SK, Kota SH (2018). A review of air quality modeling studies in India: local and regional scale. Curr Pollut Rep [Internet].

[3] Gouder C, Montefort S (2014). Potential impact of fireworks on respiratory health. Lung India [Internet].

[4] Verma C, Deshmukh DK (2014). The ambient air and noise quality in India during diwali festival: a review. Recent Res Sci Technol [Internet].

[5] Makri A, Stilianakis NI (2008). Vulnerability to air pollution health effects. Int J Hyg Environ Health [Internet].

[6] Steinhauser G, Sterba JH, Foster M, Grass F, Bichler M (2008). Heavy metals from pyrotechnics in New Years Eve snow. Atmospheric Environ [Internet].

[7] Kulshrestha UC, Rao TN, Azhaguvel S, Kulshrestha MJ (2004). Emissions and accumulation of metals in the atmosphere due to crackers and sparkles during Diwali festival in India. Atmospheric Environ [Internet].

[8] Betha R, Balasubramanian R (2013). Particulate emissions from commercial handheld sparklers: evaluation of physical characteristics and emission rates. Aerosol Air Qual Res [Internet].

[9] Hirai K, Yamazaki Y, Okada K, Furuta S, Kubo K (2000). Acute eosinophilic pneumonia associated with smoke from fireworks. Intern Med [Internet].

[10] Sharma S, Nayak H, Lal P (2015). Post-Diwali morbidity survey in a resettlement colony of Delhi. Indian J Burns [Internet].

[11] Nasir UP, Brahmaiah D (2015). Impact of fireworks on ambient air quality: a case study. Int J Environ Sci Technol [Internet].

[12] Barman SC, Singh R, Negi MP, Bhargava SK (2008). Ambient air quality of Lucknow City (India) during use of fireworks on Diwali Festival. Environ Monit Assess [Internet].

[13] Ravindra K, Mor S, Kaushik CP (2003). Short-term variation in air quality associated with firework events: a case study. J Environ Monit.

[14] Chatterjee A, Sarkar C, Adak A, Mukherjee U, Ghosh SK, Raha S (2013). Ambient air quality during Diwali Festival over Kolkata - a mega-city in India. Aerosol Air Qual Res [Internet].

[15] Ambade B, Ghosh S (2013). Characterization of PM10 in the ambient air during Deepawali festival of Rajnandgaon district, India. Nat Hazard [Internet].

[16] Khaparde VV, Pipalatkar PP, Pustode T, Rao CV, Gajghate DG (2012). Influence of burning of fireworks on particle size distribution of PM_10_ and associated barium at Nagpur. Environ Monit Assess [Internet].

[17] Perrino C, Tiwari S, Catrambone M, Torre SD, Rantica E, Canepari S (2011). Chemical characterization of atmospheric PM in Delhi, India, during different periods of the year including Diwali festival. Atmospheric Pollut Res [Internet].

[18] Deka P, Hoque RR (2014). Diwali fireworks: early signs of impact on PM_10_ properties of rural Brahmaputra Valley. Aerosol Air Qual Res [Internet].

[19] Pervez S, Chakrabarty RK, Dewangan S, Watson JG, Chow JC, Matawle JL (2016). Chemical speciation of aerosols and air quality degradation during the festival of lights (Diwali). Atmospheric Pollut Res [Internet].

[20] Sarkar S, Khillare PS, Jyethi DS, Hasan A, Parween M (2010). Chemical speciation of respirable suspended particulate matter during a major firework festival in India. J Hazard Mater [Internet].

[21] Kim KH, Kabir E, Jahan SA (2018). Airborne bioaerosols and their impact on human health. J Environ Sci (China) [Internet].

[22] Garaga R, Avinash CK, Kota SH (2018). Seasonal variation of airborne allergenic fungal spores in ambient PM_10_—a study in Guwahati, the largest city of north-east India. Air Qual Atmosphere Health [Internet].

[23] Mihdhir AA, Assaeedi AS, Abulreesh HH, Osman GE (2016). Detection, identification and characterization of some heavy metals tolerant bacteria. J Microb Biochem Technol [Internet].

[24] Sharma V, Joshi B (2010). Assessment of noise pollution during Deepawali festival in a small township of Haridwar City of Uttarakhand, India. Environ.

[25] Pulikesi M, Karthikeyan P, Sai R, Ramamurthi V, Sivanesan S (2006). Exceedences of noise level during festival day – Diwali [Internet].

[26] Pathak B, Biswas J, Bharali C, Bhuyan PK (2015). Short term introduction of pollutants into the atmosphere at a location in the Brahmaputra Basin: a case study. Atmospheric Pollut Res [Internet].

[27] Gollapalli M, Kota SH (2018). Methane emissions from a landfill in north-east India: Performance of various landfill gas emission models. Environ Pollut [Internet].

[28] (2003). Guidelines for ambient air quality monitoring [Internet].

[29] Ventura LM, Mateus VL, de Almeida AC, Wanderley KB, Taira FT, Saint'Pierre TD, Gioda A (2017). Chemical composition of fine particles (PM_2.5_): watersoluble organic fraction and trace metals. Air Qual Atmosphere Health [Internet].

[30] (1989). Standard methods for the examination of water and wastewater.

[31] Wang Y, Zhuang G, Xu C, An Z (2007). The air pollution caused by the burning of fireworks during the lantern festival in Beijing. Atmospheric Environ [Internet].

[32] Tao J, Zhang L, Engling G, Zhang R, Yang Y, Cao J, Zhu C, Wang Q, Luo L (2013). Chemical composition of PM_2.5_ in an urban environment in Chengdu, China: Importance of springtime dust storms and biomass burning. Atmospheric Res [Internet].

[33] Kerminen VM, Hillamo R, Teinila K, Pakkanen T, Allegrini I, Sparapani R (2001). Ion balances of sizeresolved tropospheric aerosol samples: implications for the acidity and atmospheric processing of aerosols. Atmospheric Environ [Internet].

[34] Cao JJ, Lee SC, Zhang XY, Chow JC, An ZS, Ho KF, Watson JG, Fung K, Wang YQ, Shen ZX (2005). Characterization of airborne carbonate over a site near Asian dust source regions during spring 2002 and its climatic and environmental significance. J Geophys Res: Atmospheres [Internet].

[35] (2004). Risk assessment guidance for superfund. Human health evaluation manual (part E, supplemental guidance for dermal risk assessment) [Internet].

[36] (1999). Guidance for conducting risk assessments and related risk activities for the DOE-ORO environmental management program [Internet].

[37] Gupta A, Joia J, Sood A, Sood R, Sidhu C, Kaur G (2016). Microbes as potential tool for remediation of heavy metals: a review. J Microb Biochem Technol [Internet].

[38] Tandon A, Yadav S, Attri AK (2008). City-wide sweeping a source for respirable particulate matter in the atmosphere. Atmospheric Environ [Internet].

[39] Moreno T, Querol X, Alastuey A, Minguillon MC, Pey J, Rodriguez S, Miro JV, Felis C, Gibbons W (2007). Recreational atmospheric pollution episodes: inhalable metalliferous particles from firework displays. Atmospheric Environ [Internet].

[40] Seinfeld JH, Pandis SN (2016). Atmospheric chemistry and physics: from air pollution to climate change.

[41] Atkinson RW, Cohen A, Mehta S, Anderson HR (2012). Systematic review and meta-analysis of epidemiological time-series studies on outdoor air pollution and health in Asia. Air Qual Atmosphere Health [Internet].

[42] Sahu SK, Kota SH (2017). Significance of PM_2.5_ air quality at the Indian capital. Aerosol Air Qual Res..

[43] Vecchi R, Bernardoni V, Cricchio D, D'Alessandro A, Fermo P, Lucarelli F, Nava S, Piazzalunga A, Valli G (2008). The impact of fireworks on airborne particles. Atmospheric Environ [Internet].

[44] Tsai HH, Chien LH, Yuan CS, Lin YC, Jen YH, Ie IR (2012). Influences of fireworks on chemical characteristics of atmospheric fine and coarse particles during Taiwan's Lantern Festival. Atmospheric Environ [Internet].

[45] Tian YZ, Wang J, Peng X, Shi GL, Feng YC (2014). Estimation of the direct and indirect impacts of fireworks on the physicochemical characteristics of atmospheric PM_10_ and PM_2.5_. Atmospheric Chem Phys [Internet].

[46] Drewnick F, Hings SS, Curtius J, Eerdekens G, Williams J (2006). Measurement of fine particulate and gasphase species during the New Year's fireworks 2005 in Mainz, Germany. Atmospheric Environ [Internet].

[47] Chaturvedi M, Tiwari R, Kulshrestha U (2017). Atmospheric reactive nitrogen fluxes and scavenging through wet deposition Over Mathura (India). Journal of Indian Geophysical Union. [Internet].

[48] Kudesia VP, Tiwari TN (1994). Noise pollution and its control.

