# Supplementary material for: Characterization of PM10 and Impact on Human Health During the Annual Festival of Lights (Diwali)
Source: J Health Pollut. 2018 Dec 1;8(20):181206. doi: 10.5696/2156-9614-8.20.181206 (PMC6285675; doi:10.5696/2156-9614-8.20.181206)
Supplement: Supplementary file 1 [file hapn-8-20-181206_s01.doc]

**Supplemental Material**

**Characterization of PM_10_ and its impact on human health during annual festival of lights (Diwali) in a residential campus**

**Rajyalakshmi Garaga^a^**[**^*^**](mailto:rajig92@gmail.com?subject=Corresponding Author) **and Sri Harsha Kota^b^**

^a*^Department of Civil Engineering, Indian Institute of Technology Guwahati, India

^b^Department of Civil Engineering, Indian Institute of Technology Delhi, India

**Health survey form**

**
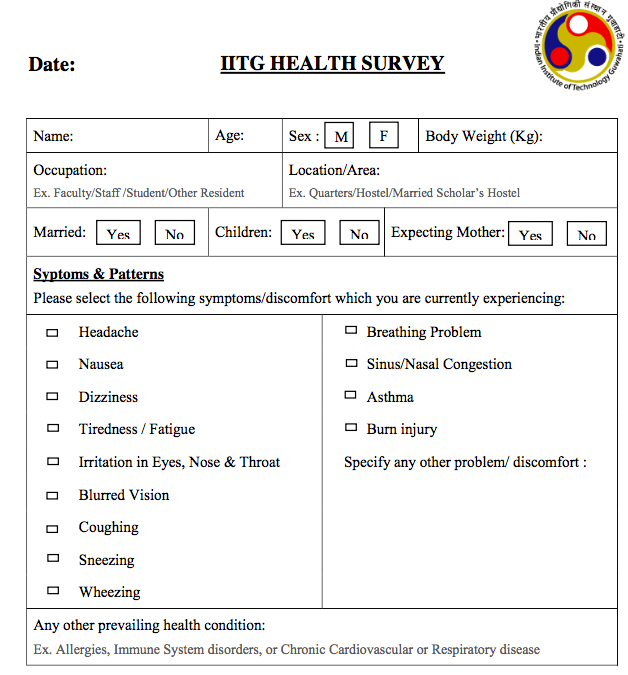
**
